# An Analysis of Arrays with Irregular Apertures in MEMS Smart Glasses for the Improvement of Clear View †

**DOI:** 10.3390/mi16020176

**Published:** 2025-01-31

**Authors:** Roland Donatiello, Mustaqim Siddi Que Iskhandar, Md Kamrul Hasan, Philipp Kästner, Muhammad Hasnain Qasim, Jiahao Chen, Shilby Baby, Basma Elsaka, Guilin Xu, Hartmut Hillmer

**Affiliations:** 1 Institute of Nanostructure Technologies and Analytics (INA) and Center for Interdisciplinary Nanostructure Science and Technology (CINSaT), University of Kassel, Heinrich-Plett-Str. 40, 34132 Kassel, Germany; kamrul.hasan@ina.uni-kassel.de (M.K.H.); kaestner@ina.uni-kassel.de (P.K.); qasim@ina.uni-kassel.de (M.H.Q.); chen@ina.uni-kassel.de (J.C.); elsaka@ina.uni-kassel.de (B.E.); hillmer@ina.uni-kassel.de (H.H.); 2 Nanoscale Glasstec GmbH, Heinrich-Plett-Str. 40, 34132 Kassel, Germany; mustaqim.iskhandar@nanoscale-glasstec.com (M.S.Q.I.); shilby.baby@nanoscale-glasstec.com (S.B.); guilin.xu@nanoscale-glasstec.com (G.X.)

**Keywords:** micromirror arrays, smart glass, improved clear view, irregular apertures, light modulation, energy saving

## Abstract

An innovative glass substrate surface technology including integrated micro-electro-mechanical systems (MEMS) is presented as an advanced light modulation, heat control, and energy management system. This smart technology is based on millions of metallic micromirrors per square meter fabricated on the glass surface, which are arranged in arrays and electrostatically actuated. The smart window application exploits an elaborate MEMS glass technology for active daylight steering and energy management in buildings, enabling energy saving, CO_2_ emission reduction, a positive health impact, and improved well-being. When light interacts with a glass substrate that has regular, repetitive patterning at the microscopic scale on its surface, these microstructures can cause the diffraction of the transmitted light, resulting in the potential deterioration of the view quality through the smart glass. A reduction in optical artifacts for improved clear view is presented by using irregular geometric micromirror apertures. Several non-periodic, irregular micromirror aperture designs are compared with corresponding periodic regular designs. For each considered aperture geometry, the irregular array reveals a reduction in optical artifacts and, therefore, by far a clearer view than the corresponding regular array. A systematic and comprehensive study was conducted through design, simulation, technological fabrication, experimental characterization, and analysis.

## 1. Introduction

Micro-electro-mechanical systems (MEMS) play an important role in a wide range of applications, including data- and telecommunication, biomedical devices, sensors, actuators, displays, and space instruments. Surface technology on a glass substrate is very rare in the MEMS world, which is dominated by silicon. However, MEMS glass substrate technology reveals great innovation potential and allows unconventional and valuable smart glass technologies.

The adoption of innovative technologies is as topical as the improvement of energy efficiency and the use of sustainable materials. Buildings and construction play a crucial part in global climate change, both as part of the problem and part of the solution. In 2022, 21% of greenhouse gas emissions and 37% of carbon dioxide (CO_2_) emissions were generated by buildings [1], both residential and commercial, which are also responsible for one-third of global energy demand [2]. A reduction in emissions during the entire life cycle of buildings can be achieved with the use of smart windows that offer numerous advantages in terms of energy efficiency but also environmental comfort, improved productivity, and well-being [3,4,5,6].

Systems and smart window solutions such as light shelves outside on the façade; Venetian sun blinds inside the room; photochromic, thermochromic, electrochromic, and gasochromic technologies; Polymer Dispersed Liquid Crystals (PDLCs); and Suspended Particle Devices (SPDs) are already on the market. Each of those technologies has advantages, shortcomings, and limitations arising from factors such as the climatic zone of use, light transmission, energy consumption, reaction time to different lighting conditions, materials employed, and user needs [7,8,9,10,11,12,13].

**Figure 1:**
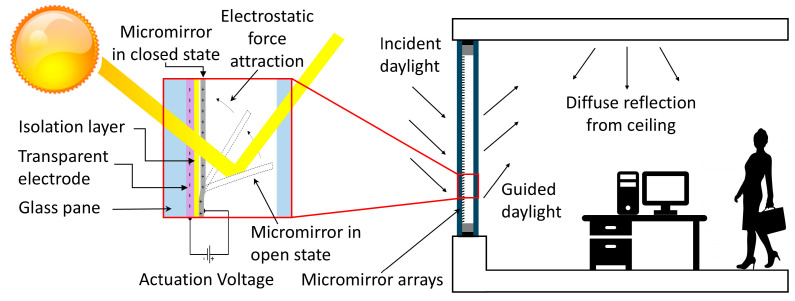
The working principle of the MEMS micromirror array for smart window applications.

Our smart window solution is an evolved concept based on smart glass substrates for daylight steering in building environments by the active control of reflection on the metallic micromirror surfaces to provide customized sunlight based on the user’s activity and the position of the sun (Figure 1) [14,15,16]. Similar transmissive applications such as smart glass or daylighting with similar actuatable microstructures can also be found in the form of microshutter arrays [17,18,19,20,21,22] or microblinds [23]. Despite having comparable arrangements, these structures utilize a curling–uncurling movement via electrostatic actuation and, therefore, have no daylight steering function.

**Figure 2:**
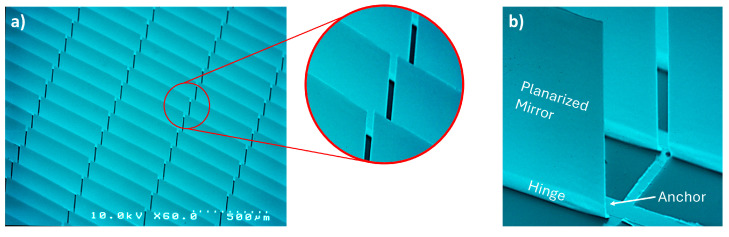
SEM micrographs of (**a**) periodic rectangular and planarized micromirror arrays; (**b**) micromirror in non-actuated state (initial open state). Modified figure from [15] with permission of Leuze publisher.

The MEMS array-based smart glass consists of millions of rectangular and planarized metallic micromirrors per square meter acting as a first electrode (Figure 2a), fabricated on an insulating layer of SiO_2_ covering a layer of transparent conductive oxide (TCO) acting as counter electrode. Each individual micromirror is composed of a fixed anchor, a bent hinge achieved through tailored stress in the thin metal layers, and a planarized micromirror surface established by localized stress compensation (Figure 2b).

**Figure 3:**
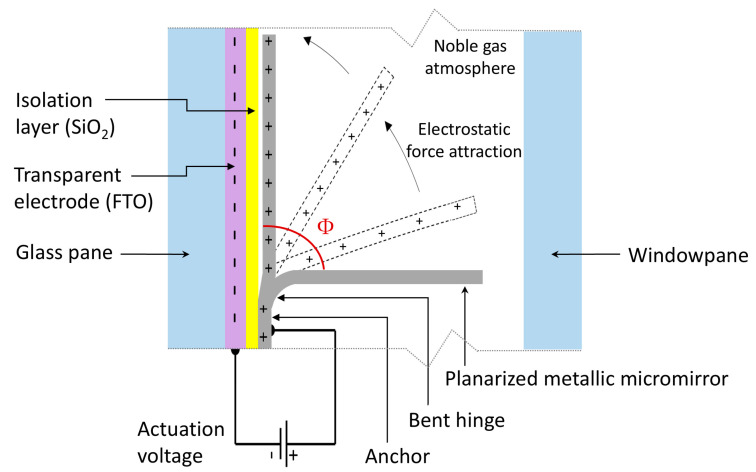
A schematic illustration in a profile view of a single micromirror, showing the working principle, the main system components, and the tilt angle Φ.

An FTO-coated glass pane is used as a substrate and subsequently housed in a noble gas atmosphere within the windowpane to protect the system from oxidation and humidity, dust, and adverse weather conditions. In the absence of an applied electrical voltage, the micromirrors are in an initial open position at a tilt angle Φ of 90° to the glass surface (Figure 3).

As the electrical voltage is increased, the micromirrors move toward the glass surface by virtue of the electrostatic force attraction until they close. Before a critical pull-in point is reached, any position in between is possible due to the balance between the electrostatic force attraction caused by the applied voltage and the elastic counter force in the bent hinge of the micromirrors, allowing the steering of sunlight. The typical pull-in voltage point in our group is in the range of 25–50 V, depending on the design and fabrication process used. Due to the elastic restoring moment defined by the intrinsic stress in the hinge, the micromirrors return to an upright position when the electric voltage is turned off.

An intelligent sensing system gathers data by monitoring the brightest spot in the sky, the user movement within the room, and the temperature both inside and outside. These data are transmitted to the control unit, which adjusts the functionality of the micromirror arrays as needed by the user in the room.

Potential energy savings were analyzed using a self-developed universal simulation tool (“SAVINGS”), which compares the performance of the MEMS smart window to conventional window blinds. This tool can be applied globally wherever meteorological data are available. The results indicate that the MEMS smart window can achieve up to 35% energy savings for heat management and lighting [13,15].

Our smart glass system therefore works in transmission, with groups of neighboring micromirrors (subfields) that can be oriented together and in the same way to transmit light through the glass and steer it to the desired area. In contrast, one of the world’s most commercially successful MEMS devices, the Digital Light Processing (DLP) of Texas Instruments Corporation, works purely with the reflection of light. At the heart of the DLP technology is the Digital Micromirror Device (DMD) chip, which consists of an array of individually addressable silicon micromirrors (pixels in projector applications), the entire control electronics and micromechanics of which are made on a standard Complementary Metal-Oxide Semiconductor (CMOS) wafer [24]. Each micromirror allows the steering of reflected light within microseconds by exploiting electrostatic activation and has two mechanical stops. This means that it can remain in only two angular positions, e.g., +10° and −10°. This technology obviously cannot be used in applications such as smart windows because silicon does not allow the transmission of visible light through the mirror plane (substrate plane). In addition, silicon is also excluded for cost reasons for large-area arrangements.

**Figure 4:**
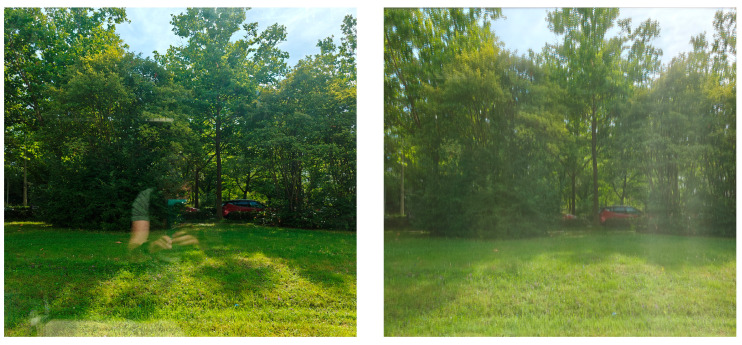
(**left**) The view through a standard double pane window of our institute. (**right**) The view through our fabricated 30 × 30 cm^2^ MEMS smart glass with periodic rectangular micromirrors. Both pictures were taken on the same day and at the same time, thus with the same daylight intensity and sun position.

The miniaturization of micromirrors is crucial to offer a fast response and low power consumption and to minimize material fatigue, thus ensuring a long lifetime and high mechanical stability [25]. In addition, the micromirrors are not noticeable to the human eye at distances of more than 25 cm. On the other hand, the dimensions of the transparent areas (apertures) and metallic structure of the MEMS array are in the order of magnitude of the visible light wavelength, and this, together with its periodicity, may lead to visible optical artifacts through our fabricated smart glasses due to diffraction phenomena (Figure 4).

An improved clear view through the smart window can be achieved by using micromirror arrays with non-periodic and irregular geometry, as proposed in our patent [26], realizing as few parallel or equidistant structures as possible and having a laterally varying duty cycle within the array. In the sequel, the fundamentals of the diffraction grating phenomenon and the connection with the Fourier transform as well as the geometries of the periodic and non-periodic micromirror arrays studied will be introduced, followed by chapters presenting the results of the simulations and experiments conducted with related discussions. Then, there will be a chapter dedicated to the fabrication of the novel non-periodic geometry of a micromirror array and, finally, the conclusion.

## 2. Fundamentals: Periodic and Non-Periodic Arrays

### 2.1. Grating Diffraction

Periodic micromirror arrays on glass substrates can be regarded as 3D periodic amplitude transmission gratings from a diffraction observation point of view, which is a three-dimensional structure that transmits light through regularly spaced and identically dimensioned transparent apertures separated by opaque spacings of identical size. The transparent geometric parts and the sharp-edged opaque regions create a variation in the intensity of the transmitted light, which further changes when the micromirrors are actuated in accordance with their elevation in the third dimension. This, in turn, leads to the variation in the grating duty cycle (ratio of opaque parts to transparent parts), while the grating periods remain constant.

**Figure 5:**
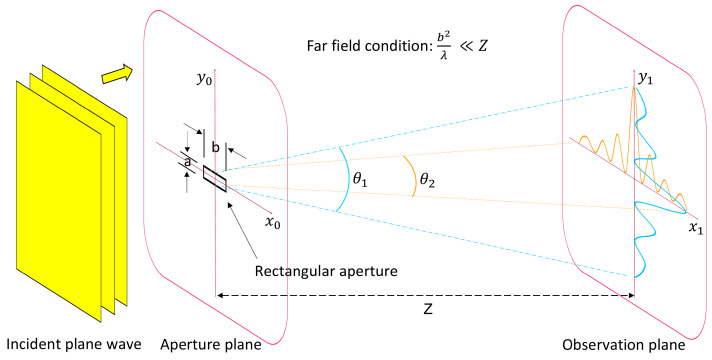
An illustration of light diffraction at a 2D rectangular aperture (transparent part in the aperture plane) decomposed into the associated 1D diffraction patterns in the observation plane in the far field, simplified for incident plane waves and visible light propagation perpendicular to the wavefronts. The diffraction fringe spacing is narrower along the *x*_1_ axis (in orange) and wider along the *y*_1_ axis (in blue) because a<b in the rectangular aperture.

The diffraction occurs due to the periodic modulation of the wave’s amplitude when visible light with wavelength *λ* passes through the periodic structure of the micromirror array with specific dimensions in the range between about *λ*/10 and 100*λ*. Each point inside a transparent aperture creates wavelets (secondary elementary waves), which spread out in all directions with the same frequency as the primary wave according to Huygens’ principle [27]. The wavelets interfere constructively or destructively at different angles, forming a diffraction pattern in the observation plane that might lead to a reduction in the clear view through the smart window. Fraunhofer diffraction is considered for the smart window application because the distance between the light source (the sun) and the grating, as well as the distance between the grating and the observation plane (the user in the room), is very large compared to the size of the apertures. The diffraction pattern on the observation plane is characterized by an intense and broad central maximum and less intense secondary maxima at the sides, alternating with intensity minima (Figure 5).

The Fraunhofer diffraction pattern of an aperture in a far-field regime can be charact1erized by the Fourier transform of the aperture function. The propagation of light through gratings in the absence of magnetic materials can be described by the Helmholtz equation:

(1) $$\nabla^{2}\vec{E}=\mu_{0}{\;\varepsilon }_{0}\frac{\partial^{2}\vec{E}}{{\partial t}^{2}}\;$$

where

*µ*_0_ = magnetic permeability of free space;
ε_0_ = electric permittivity of free space.

Given that the coordinates in the plane of the aperture are *x*_0_ and *y*_0_, the coordinates in the plane of observation are *x*_1_ and *y*_1_. Given that both planes are perpendicular to the z-direction and placed at a distance from each other of *Z* when the light propagates uniformly and without disturbance along the *z* axis, the solution of the Helmholtz equation for plane waves is of the form:

(2) $$\vec{E}\left(z,t\right)={\vec{E}}_{0}e^{i(k_{z}z-\omega t)}$$

where

*E*_0_ = electrical field amplitude of the wave (V/m);
*k* = wave number = $\frac{2\pi }{\lambda }$ = $\frac{\omega }{c}$ (1/m);
*ω* = angular frequency = 2πν (radians/s);
*ν* = frequency (Hz).

In far-field conditions, the Fraunhofer diffraction formula can be expressed as follows:

(3) $$E\left(x_{1},y_{1}\right)\propto \int_{-\infty }^{\infty }\int_{-\infty }^{\infty }\;exp\frac{-i2\pi }{z\lambda }(x_{0}x_{1}+y_{0}y_{1}){\;A\left(x_{0},y_{0}\right)E\left(x_{0},y_{0}\right)dx}_{0}{dy}_{0}$$

Equation (3) shows that the electrical field distribution $E\left(x_{1},y_{1}\right)$ on the observation plane is proportional to the Fourier transform of the aperture function $A\left(x_{0},y_{0}\right)$, which describes all transparent parts of the grating on the aperture plane [27]. In terms of k-vector components, Equation (3) can be rewritten as follows:

(4) $$E\left(k_{x1},k_{y1}\right)\propto \int_{-\infty }^{\infty }\int_{-\infty }^{\infty }\;exp-i(x_{0}k_{x1}+y_{0}k_{y1}){\;A\left(x_{0},y_{0}\right)E\left(x_{0},y_{0}\right)dx}_{0}{dy}_{0}$$

where *k_x_*_1_ and *k_y_*_1_ are the conjugate variables of *x*_0_ and *y*_0_ and have respectively reciprocal units [28]:

(5) $$k_{x1}=\frac{2\pi x_{1}}{\lambda z};\;k_{y1}=\frac{2\pi y_{1}}{\lambda z}$$

### 2.2. Studied Geometrical Micromirror Apertures

To control the phenomenon of diffraction, techniques such as the modulation and modification of geometric structures are used in optics, especially to suppress or shift unwanted diffraction orders or to improve the performance of devices such as diffraction gratings, waveguides, or optical nanostructures. Modifying the geometry of structures that cause diffraction, such as apertures or slits in diffraction gratings, is a well-known approach in the scientific literature [29,30,31,32,33,34,35,36,37,38,39].

**Figure 6:**
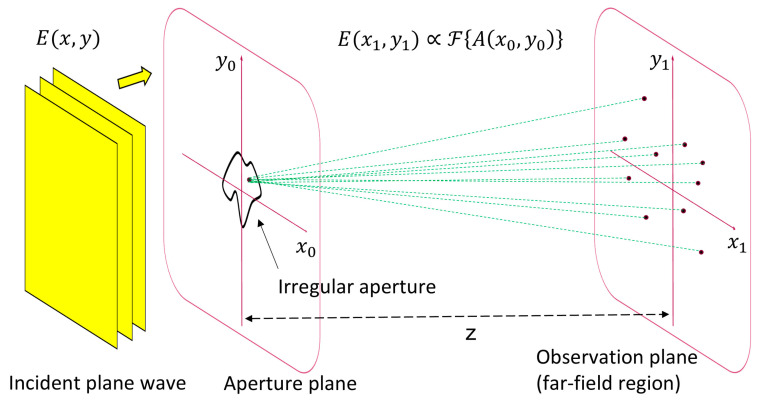
An illustration of light diffraction at an irregular aperture (transparent part in the aperture plane), simplified for plane waves. The observation plane is in the far field. The far-field electrical distribution $E(x_{1},y_{1})$ is proportional to the Fourier transform of the aperture function $A\left(x_{0},y_{0}\right)$.

Our concept aims to improve the clear view through MEMS smart glasses by using micromirror arrays with non-periodic and irregular geometries using as few parallel or equidistant structures as possible to have a laterally variable duty cycle within the array and allow the corresponding phase modulation to reduce the intensity of the higher orders of diffraction (Figure 6).

**Figure 7:**
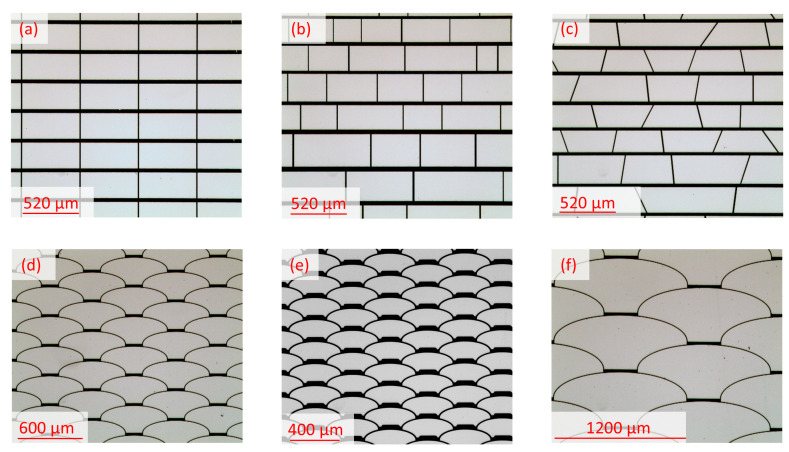
Optical micrographs of studied geometries: (**a**) periodic (or regular) rectangular; (**b**) non-periodic (or irregular) rectangular; (**c**) non-periodic (or irregular) trapezoidal; (**d**) *fish skin* Medium; (**e**) *fish skin* Small; (**f**) *fish skin* Large. The opaque spacing represents the deposited metallic structure and the clear parts of the apertures of the arrays.

Various non-periodic structures were designed using apertures with different geometries. For this study, a non-periodic array with rectangular apertures (Figure 7b) and a non-periodic array with trapezoidal apertures (Figure 7c) were analyzed, the varying dimensions of which were averaged over the entire respective array for an accurate comparison with the periodic design, which features rectangular apertures of 520 µm × 200 µm in size (Figure 7a). In addition, we designed the novel non-periodic *fish skin* geometry in three different dimensions: Medium (Figure 7d); Small (Figure 7e); and Large (Figure 7f). The *fish skin* Medium configuration is comparable in aperture size range and duty cycle to the periodic rectangular array design. The diffraction produced by the periodic and non-periodic arrays was analyzed and compared both theoretically and experimentally.

## 3. Simulated Diffraction

### 3.1. Elaboration of Interpretation Tools for Fourier Transforms and Diffraction Patterns

To enable a better understanding of simulated Fourier transforms and experimental diffraction patterns, some basic 2D grating structures are considered first. This is crucial to enable, in Section 3.2 and Section 4, a systematic quantitative interpretation of the characteristic features and patterns of regular and irregular gratings.

**Figure 8:**
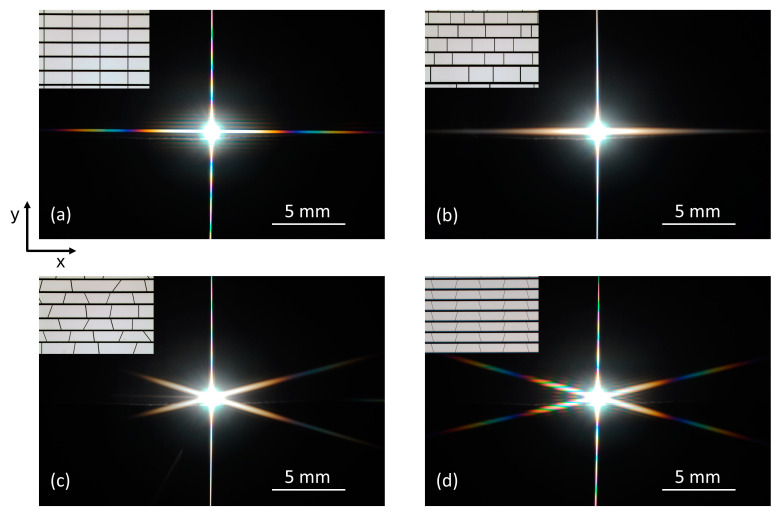
The diffraction pattern of four different gratings using white light from a tungsten lamp. Note that the measurement was performed without the white star object used below. The shapes of the gratings are as follows: (**a**) regular rectangular; (**b**) irregular rectangular; (**c**) irregular trapezoidal; (**d**) regular trapezoidal. The regular gratings were obtained by averaging all the geometrical parameters of the irregular gratings. Modified figure from [39] with the permission of Leuze publisher.

Figure 8 displays the diffraction pattern of white light from a tungsten lamp at four different 2D gratings. The most prominent feature is a “bundle of beams”-like (star-like) pattern starting always from the center of the diagram.

In the case of regular rectangular apertures (inset Figure 8a) and irregular rectangular apertures (inset Figure 8b), only two stripes are observed, oriented vertically to each other. All the rectangles are oriented in such a way that only vertical or horizontal side lines occur. Therefore, the horizontal stripe (in the x-direction) of the diffraction star originates from all the vertical side lines of the rectangular apertures in the y-direction. Analogously, the vertical stripe (in the y-direction) of the diffraction star results from all the horizontal side lines of the rectangular apertures in the x-direction.

In the case of the irregular trapezoidal aperture (inset Figure 8c) and the regular trapezoidal one (inset Figure 8d), three stripes are observed. All the trapezoids are oriented in such a way that the two parallel side lines are oriented horizontally (x-direction). Therefore, the vertical stripe (in y-direction) of the diffraction star originates from all the horizontal side lines of the rectangular apertures in the x-direction. In addition, all trapezoids reveal two angled side lines. The second and third stripes of the diffraction star are oriented vertically to these two side lines, respectively. The diffraction from regular gratings (Figure 8a,d) is much more pronounced and clearly visible by the color transitions on the stripes.

**Figure 9:**
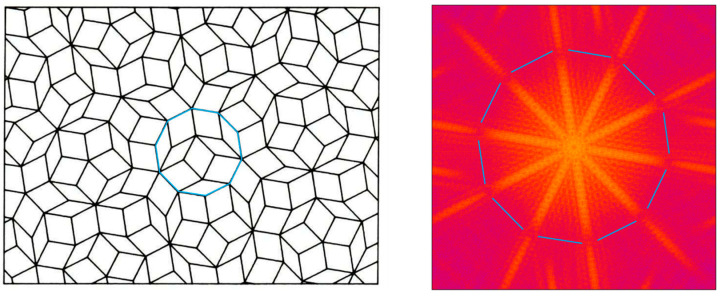
(**left**) Arabic pattern. (**right**) Corresponding Fourier transform; colors closer to yellow/white correspond to higher values of diffraction intensity.

These findings are now used for the interpretation of the simulated Fourier transform (proportional to the diffraction pattern) of a special 2D grating called an “Arabic pattern” (Figure 9left).

The Arabic pattern includes two kinds of rhombuses. The longest diagonal of the rhombuses is identical for both. Instead, the shortest diagonals occur in two versions: a short and a medium-sized one. Thus, we have the small rhombus and the large rhombus. In addition, there are five different orientations of the small rhombus and the large rhombus, respectively. The Arabic pattern has regular as well as irregular features, but only five different orientations of the rhombus side lines occur. The angle between these orientations is 71.5°. In consequence, vertical diffraction to all these five-line orientations occurs, leading to a star-like diffraction in the Fourier transform with ten stripes (Figure 9right). However, another feature is worthy of mention. The circular structure interrupting the stripes of the diffraction star (visualized by the broken blue line in Figure 9right) originates from the decagon (a polygon with 10 identical sides) shown in Figure 9left, in blue. Note that in Section 3.1, the color code is not given like in Section 3.2, intentionally, to keep it simple at the beginning.

**Figure 10:**
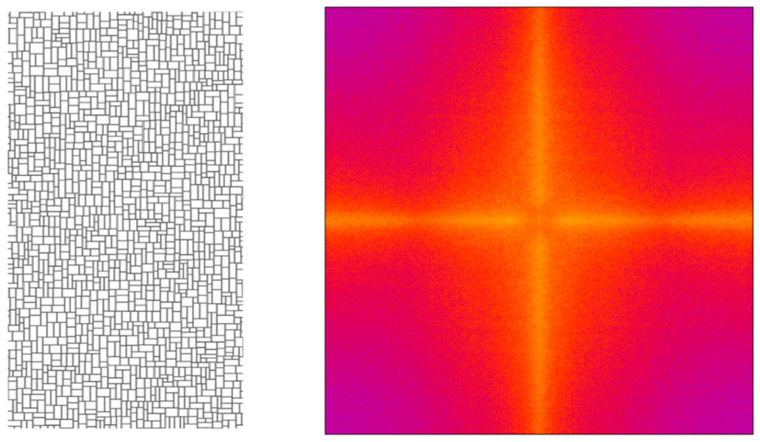
(**left**) A rectangular grating with a high degree of irregularity. (**right**) The corresponding Fourier transform; colors closer to yellow/white correspond to higher values of diffraction intensity.

Figure 10left depicts an irregular grating with rectangular apertures, which are combined in the y-direction in a more irregular way than the inset in Figure 8b. In consequence, the corresponding Fourier transform (Figure 10right) reveals much fewer diffraction features but still shows a pronounced diffraction star with stripes in two orientations, which is caused by the two occurring side line orientations of the rectangular apertures.

**Figure 11:**
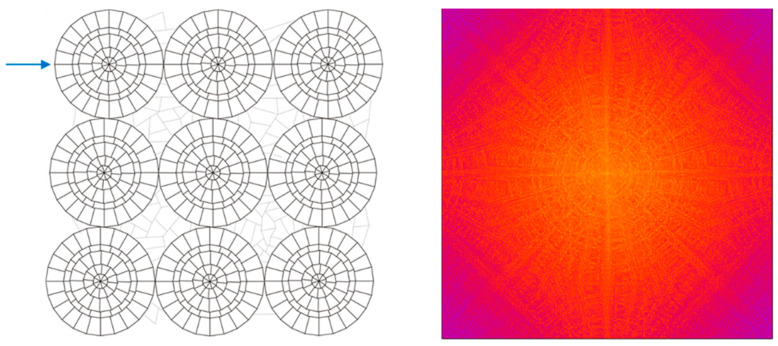
(**left**) Nine multi-ring gratings with irregular patterns in between. (**right**) Corresponding Fourier transform; colors closer to yellow/white correspond to higher values of diffraction intensity.

Finally, a structure is considered to consist of nine ring-shaped structures (Figure 11left). Each ring consists of polygons with up to 24-line parts. The apertures of the six different rings are oriented a bit irregularly. However, there is still noticeable regularity inside. The orientations of respective vertical lines to the polygons still coincide to intentionally elaborate a better understanding of simulated Fourier transforms.

A severe accumulation of nearly identical horizontal orientations is designed and indicated by a blue arrow (oriented in x-direction). This orientation causes a vertical stripe in the Fourier transform (Figure 11right), which coincides with the most visible stripe. For an outer ring, the number of differently oriented side lines of the aperture is 12. The inner rings have slightly different orientations, and the number in total is about 50. This produces a Fourier transform that shows only a star structure, which is rather blurry (Figure 11right).

The following conclusions can be drawn:

- The number of differently oriented lines corresponds to the same number of stripes in the Fourier transforms that all pass through the center. A star-like stripe bundle appears, meaning that the number of stripes occurring in the diffraction star corresponds to the number of differently oriented side lines of the apertures.
- Each individual side line orientation causes a stripe vertical to it in the Fourier transform, respectively.
- The higher the number of differently oriented lines, the less pronounced the corresponding visibility of the stripes in the Fourier transform.
- The less structure the Fourier transform reveals, the smaller seems to be the influence of diffraction.
- Irregular gratings reveal less structure in the Fourier transforms (less diffraction) than regular gratings.
- The design rule for achieving strong diffraction reduction in gratings is to include as much as possible different side line orientations of the apertures (number *p* of different orientations) into the grating. The diffraction star in the Fourier transform will have the same number *p* of differently oriented stripes. If *p* goes to infinity, the star dissolves. The corresponding apertures have no linear side lines anymore but are bordered by free forms.

Next, diffraction in the y-direction is calculated for the regular rectangular grating (Figure 7a). This is a one-dimensional problem, and Equation (6) below gives the diffraction intensity $I\left(\varphi \right)$, which will be normalized to 1 (maximum intensity $I_{0}=1)$:

(6) $$I\left(\varphi \right)=I_{0}{{\left[\frac{sin\left(\pi \frac{L_{y}}{\lambda }sin\varphi \right)}{\pi \frac{L_{y}}{\lambda }sin\varphi }\right]}^{2}\left[\frac{sin\left(N\;\pi \frac{{⋀}_{y}}{\lambda }sin\varphi \right)}{sin\left(\pi \frac{{⋀}_{y}}{\lambda }sin\varphi \right)}\right]}^{2}$$

where

λ = wavelength;
*L_y_* = aperture size (slit width);
*Λ_y_ =* grating period;
*N =* number of slits in the grating.

**Figure 12:**
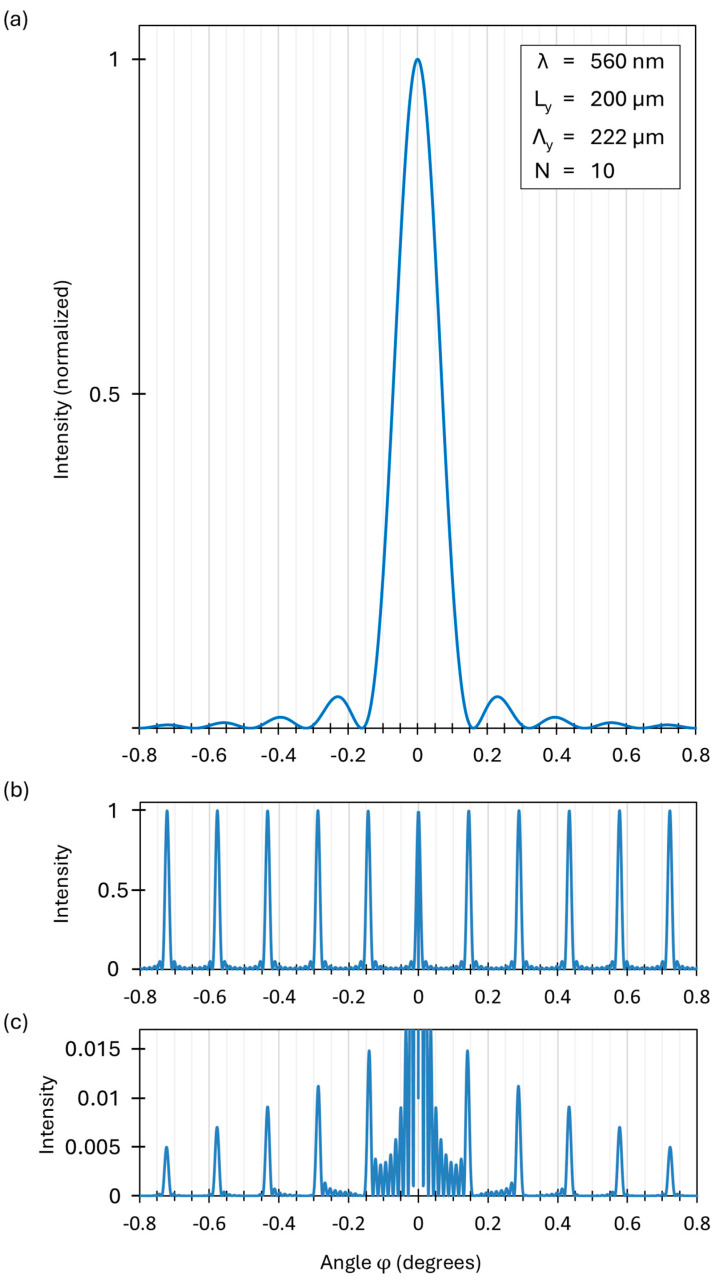
The theoretical model calculations of a one-dimensional grating based on Equation (6). (**a**) An envelope representing the diffraction at a single slit. (**b**) The grating diffraction for a slit width of almost zero. (**c**) Multiplication of both, representing the total diffraction pattern.

The term in the first bracket is squared and represents the envelope of the diffraction pattern (Figure 12a). This describes the diffraction pattern of a single slit. The term in the second bracket is also squared and describes the diffraction of the grating, with the slit width *L_y_* considered to be almost zero. This function describes the dominating main maxima and the side maxima in between (Figure 12b). If *N* slits of the grating are illuminated, then *N-2* side maxima are visible between neighboring main maxima. The larger *N* is, the smaller the linewidth of the main maxima. Ten slits (*N* = 10) are considered in Figure 12 because the lines in Figure 12b would be too sharp if *N* was as large as in reality, and the side maxima would not be visible anymore (in the diffraction experiments below, we have an *N* of about 200). Note that Figure 12 was drawn for a wavelength λ=560 nm (monochromatic light). The zero orders always come at φ=0° in all subfigures.

Figure 12c shows the whole diffraction pattern, including the square of the product of the slit diffraction envelope function (first bracket term of Equation (6)) and the grating diffraction profile (second bracket term of Equation (6)) for non-zero *L_y_* (real value). Due to the large intensity values for the envelope function for the zero order, a large number of side maxima are visible between the zero- and first-order of the envelope.

The envelope in Figure 12a shows, from the left to the right, orders 4, −3, −2, −1, 0, +1, +2, +3, +4. The grating diffraction profile for Ly ≈ 0 (almost zero) in Figure 12b shows, from the left to the right, the orders −5, −4, −3, −2, −1, 0, +1, +2, +3 +4, +5.

The diffraction experiments in Section 4 were performed with white light from a tungsten gas lamp with a spectral full width of half maximum (FWHM) of 500 nm. The first-order main maximum is broadened by the overlap of all the spectrally sharp main maxima of individual wavelengths included in the lamp spectrum. This is confirmed by Equation (6) calculating the angle φ=0.141° for the first-order main maximum for λ=560 nm, and the angle φ=0.171° for the first-order main maximum for λ=680 nm, as well as the angle φ=0.287° for the second-order main maximum for λ=560 nm, and the angle φ=0.349° for the second-order main maximum for λ=680 nm.

In conclusion, the destructive interference parts of the envelope profile significantly dampen the main maxima (Figure 12c) for its first two orders, since the angular positions of the main maxima (constructive interference) coincide nearly with the angular position of the destructive interference angles of the envelope. This is of advantage for the clear view through the MEMS smart glass. However, a further drastic reduction of the diffraction pattern is shown in Section 3.2 and Section 4 via the grating apertures of non-rectangular and non-regular geometries.

### 3.2. Investigation of K-Space Representations

**Figure 13:**
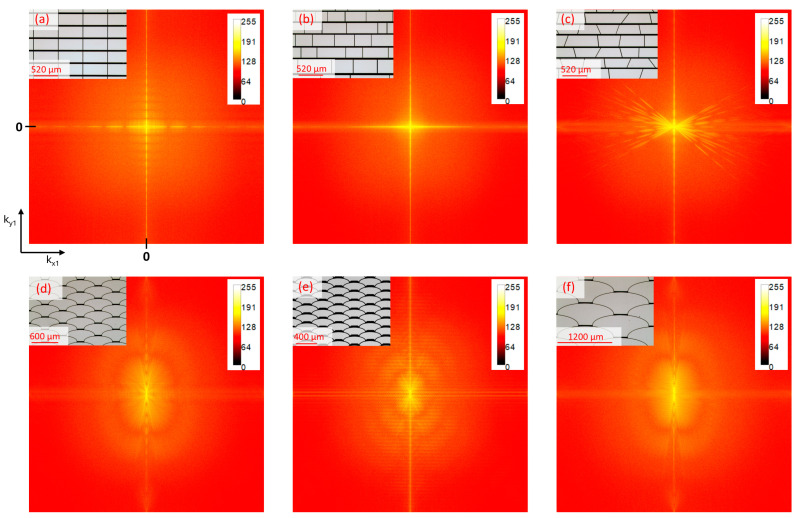
The simulated diffraction patterns of studied geometries: (**a**) periodic rectangular; (**b**) non-periodic rectangular; (**c**) non-periodic trapezoidal; (**d**) *fish skin* Medium; (**e**) *fish skin* Small; (**f**) *fish skin* Large. The opaque spacing represents the deposited metallic structure and the clear parts of the apertures of the arrays. The scale bar shows the intensity of the diffraction, higher values correspond to the colors closer to yellow/white. A modified figure from [38] with the permission of Leuze publisher.

According to the Fourier diffraction theorem described in Section 2.1, the diffraction pattern produced by an aperture in the path of light can be described using Fourier transform techniques. For this purpose, the well-known ImageJ v. 1.54i software with Numerical Propagation plug-in v. 1.2 was used, of which the integrated full set of tools enables the geometric gratings in the spatial domain (*x*_0_, *y*_0_) to be transformed into k-space representations (Figure 13) describing the related diffraction patterns in the far field in the observation plane (*x*_1_, *y*_1_).

The k-space directly influences the clarity and quality of the images produced in optical systems. In accordance with the conventional qualitative interpretation of Fourier analysis, low spatial frequencies (near the center of the k-space) contain the information on signal-to-noise ratio and image contrast, while high spatial frequencies (outer peripheral regions of k-space) contain the information that determines image resolution [40,41].

The Fourier spectra of periodic and non-periodic designs (Figure 13a–c) show a dominant vertical diffraction stripe resulting from the periodic horizontal borders of the opaque spacings and a dominant horizontal diffraction stripe resulting from the vertical borders. The design of the trapezoidal array exhibits, in addition, a star-like effect in the center, resulting from diffraction along the inclined borders. Vice versa, the arrays with *fish skin* geometry (Figure 13d–f), notably in the Medium configuration (Figure 13d), show a reduced diffraction pattern, i.e., a general lower intensity and an increased homogeneity, resulting in less distortion, an enhanced image resolution, and, thus, a significantly improved clear view through the smart glass.

## 4. Experimental Diffraction

**Figure 14:**
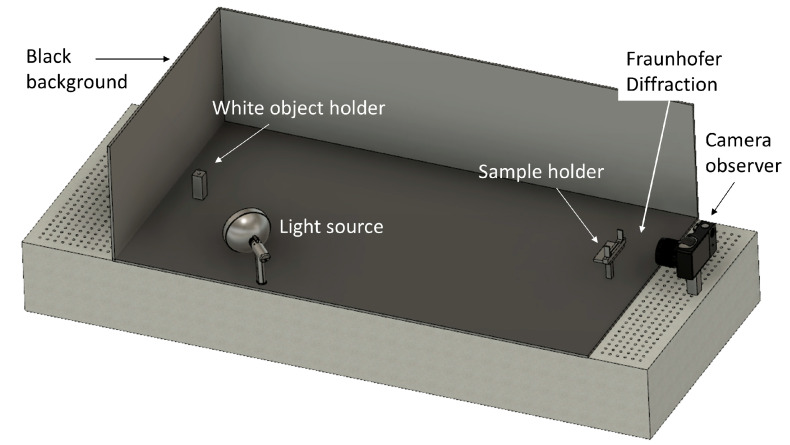
A schematic of the self-constructed Fraunhofer diffraction set-up. A tungsten-halogen lamp is used as the light source covering the whole visible spectral range to illuminate a white object during the experiment. The observer monitors and saves the images on a CCD chip of a high-quality digital camera.

Array designs were then investigated in a self-constructed set-up to acquire images for the study of related diffraction intensity profiles (Figure 14). For this purpose, each design was transferred by photolithographic process and metal deposition onto 10 × 10 cm^2^ glass substrates coated with TCO and SiO_2_ layers, resulting in the related 2D arrays that represent the metallic structure of the MEMS smart glass without micromirrors. In the first approximation, 2D arrays correspond, therefore, to 3D arrays with free-standing micromirrors in a fully open position (90° to the glass surface) for vertical light incidence.

**Figure 15:**
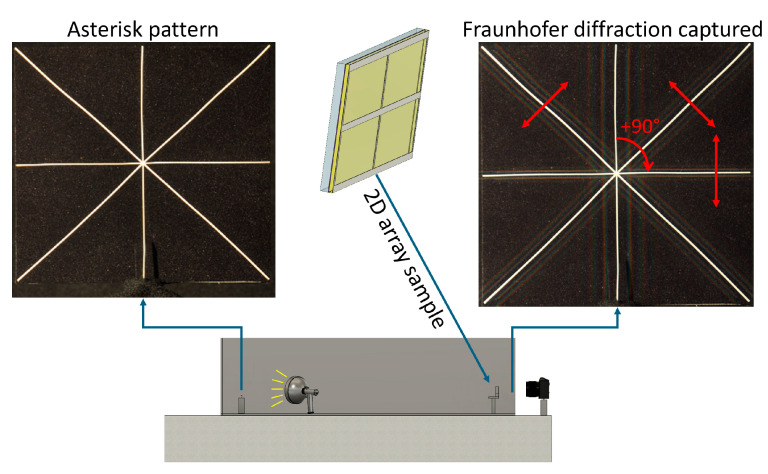
A schematic of the set-up evidencing the position of the 2D array sample. For characterization and monitoring of Fraunhofer diffraction, a white asterisk shape was used due to its clear orientations on lines with different angles of inclination (+90°, +45°, −45°). The red arrows represent the corresponding diffraction orientations analyzed (0°, −45°, +45° respectively).

The samples were then mounted one by one and aligned between the camera observer and the white object at a distance satisfying the far-field condition in the diffraction set-up located in a darkroom for stray light and noise reduction. A white asterisk shape wasused to considerably amplify the reflected light as a white object for the characterization of the diffraction patterns on lines with different angles of inclination (+90°, +45°, −45°) (Figure 15). As a note, the diffraction pattern measured for the +90° bar orientation corresponds to diffraction in the 0° direction. Several high-resolution images were taken for each sample and at different locations in the sample to ensure valid statistics.

**Figure 16:**
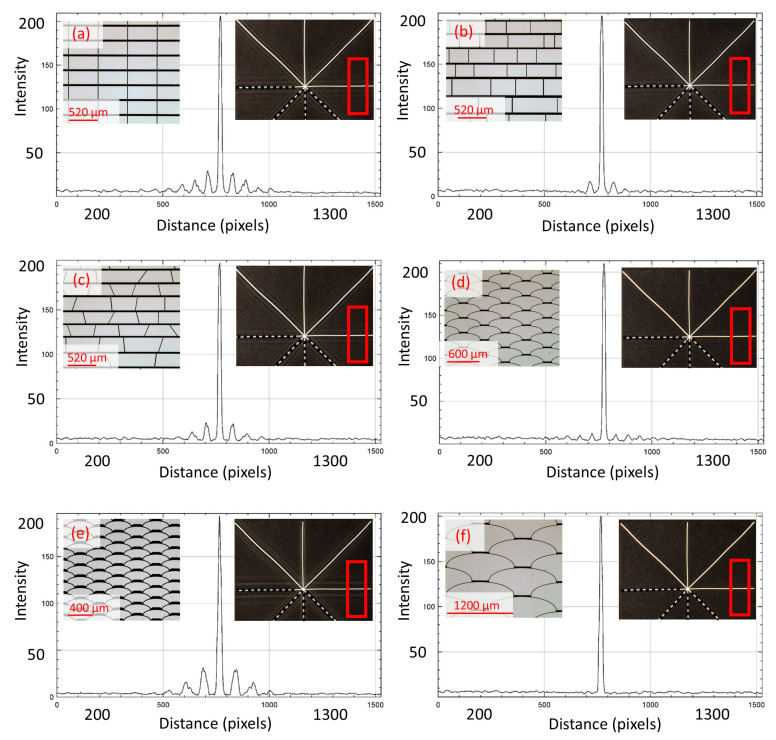
Intensity profile plots of diffraction patterns whose digital images with related designs are inserted as insets. Measurements performed on white bar with +90° orientation, corresponding to diffraction in 0° direction. The red rectangular shape represents the ROI considered.

**Figure 17:**
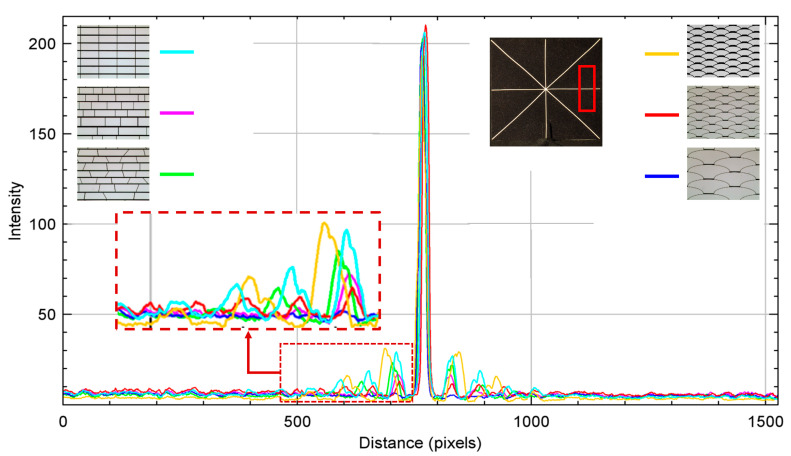
Intensity profile plot of superimposed diffraction patterns on white bar with +90° orientation, corresponding to diffraction in 0° direction. Related designs are inserted as insets. The red rectangular shape represents the ROI considered.

**Figure 18:**
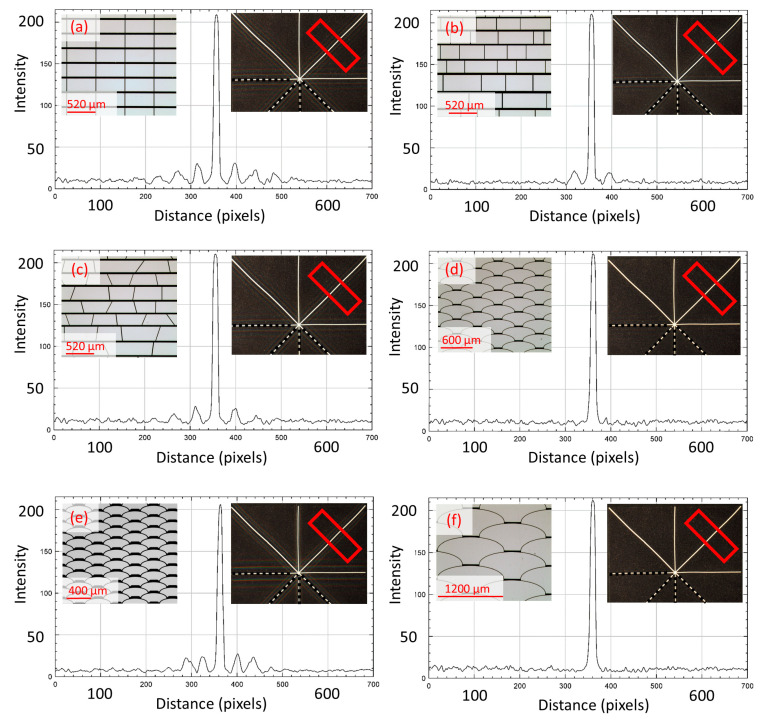
Intensity profile plots of diffraction patterns whose digital images with related designs are inserted as insets. Measurements performed on white bar with +45° orientation, corresponding to diffraction in −45° direction. The red rectangular shape represents the ROI considered.

**Figure 19:**
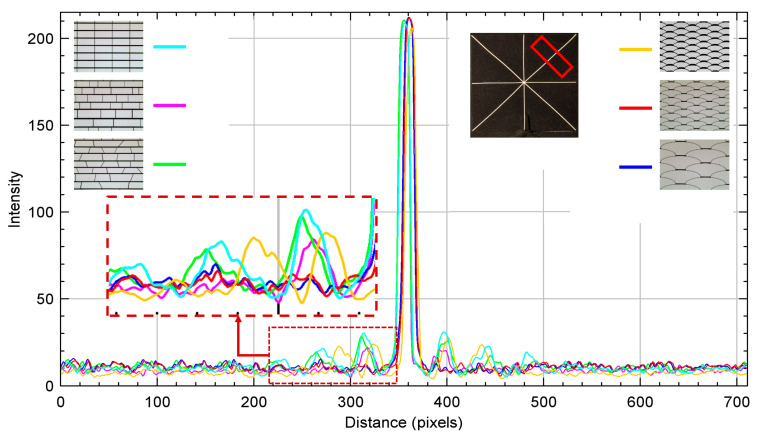
Intensity profile plot of superimposed diffraction patterns on white bar with +45° orientation, corresponding to diffraction in −45° direction. Related designs are inserted as insets. The red rectangular shape represents the ROI considered.

**Figure 20:**
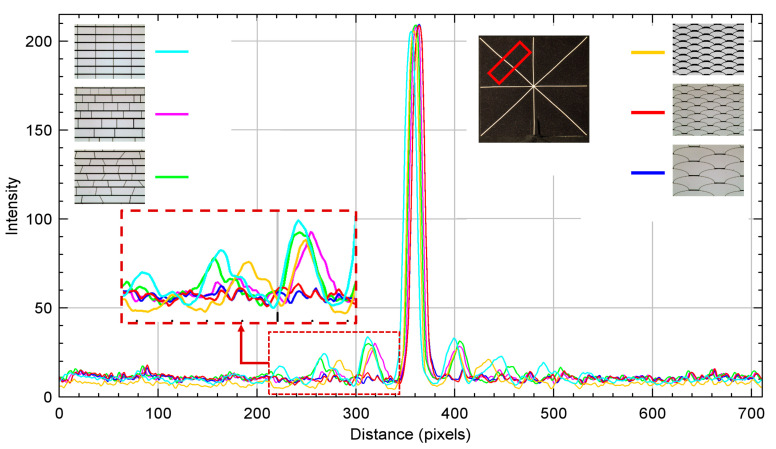
Intensity profile plot of superimposed diffraction patterns on white bar with −45° orientation, corresponding to diffraction in +45° direction. Related designs are inserted as insets. The red rectangular shape represents the ROI considered.

The pictures were then processed to measure and evaluate the corresponding diffraction intensity profiles with the ImageJ software, for which a rectangular ROI (Region of Interest) of the same size and in the same positions on each bar of the asterisk shape was used in results in the following Figure 16, Figure 17, Figure 18, Figure 19 and Figure 20.

The measurements along the bar with an inclination angle of +90° show lower non-zero orders of diffraction intensity for the array based on the *fish skin* Medium geometry (Figure 16d) compared with the non-periodic rectangular (Figure 16b) and non-periodic trapezoidal (Figure 16c) designs and significantly lower non-zero orders of diffraction intensity than periodic rectangular design (Figure 16a) if they are similar in aperture size and duty cycle.

Diffraction is observed at the smaller apertures of the *fish skin* geometry (Figure 16e) since they are closer in order of magnitude to the visible wavelengths of light than the larger *fish skin* apertures (Figure 16d,f). This confirms the simulation results, i.e., minimum parallel lines and free forms are the best option because there are inevitably far fewer boundary lines parallel to the aperture. This further conclude that the novel *fish skin* geometry is the winning solution for MEMS smart glass arrays, leading to a noticeable improvement of the clear view. The representation of the results in a single graph offers a quick comparison of the non-zero diffraction orders generated by the geometries under analysis and shows how the intensity profile of Fraunhofer diffraction results in a significant portion of the light energy being concentrated in the zero- and first-order maxima (Figure 17).

Analyses on the diffraction patterns for bar orientations at +45° and −45° further corroborate that the array with *fish skin* Medium geometry has significantly reduced non-zero orders of diffraction intensity compared to the rectangular periodic design and the other non-periodic arrays (Figure 18, Figure 19 and Figure 20). As expected, *fish skin* Small geometry confirms the intensities of non-zero orders of diffraction comparable to those of periodic and non-periodic rectangular arrays in addition to the non-periodic trapezoidal array due to the size of its apertures being closer to visible light wavelengths than the *fish skin* Medium configuration.

**Figure 21:**
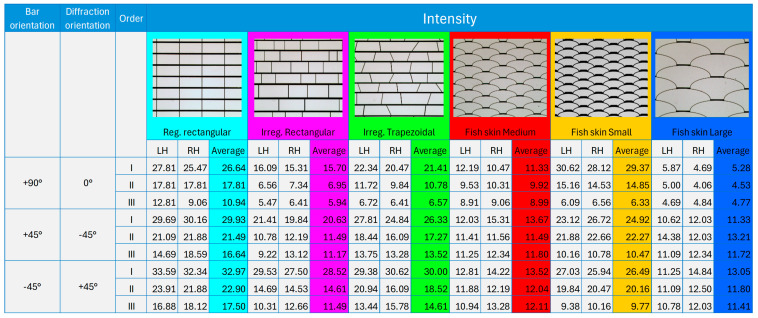
Result of the intensity peaks of the first 3 diffraction orders (I, II, III). In the highlights are the geometries and the corresponding values calculated as an average between negative (LH) and positive (RH) orders. The colors used in the previous intensity plots have been reproduced to represent the related geometries.

The intensity distributions of the first three diffraction orders are elaborated for the three orientations considered (+90°, +45°, −45°) and for each geometry studied (Figure 21). The values represent the peak intensity of the negative (LH) and positive (RH) diffraction orders and the average of the negative and positive values (Average).

**Figure 22:**
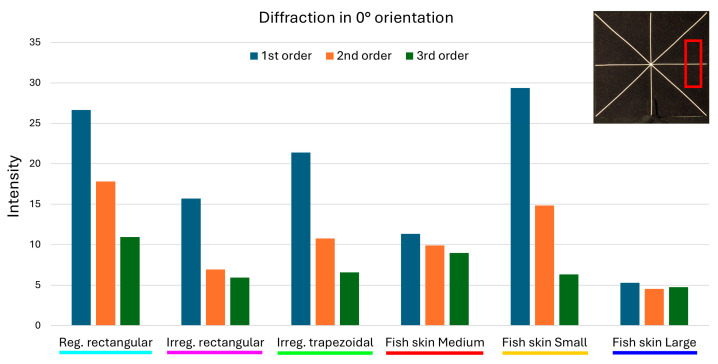
A comparison of the intensities of the first 3 diffraction orders in the 0° direction. The red rectangular shape represents the ROI considered.

**Figure 23:**
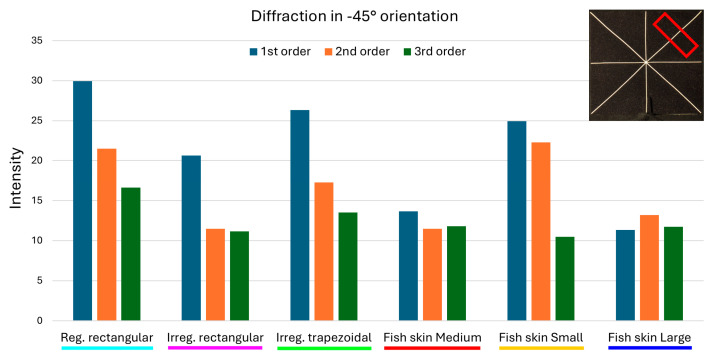
A comparison of the intensities of the first 3 diffraction orders in the −45° direction. The red rectangular shape represents the ROI considered.

**Figure 24:**
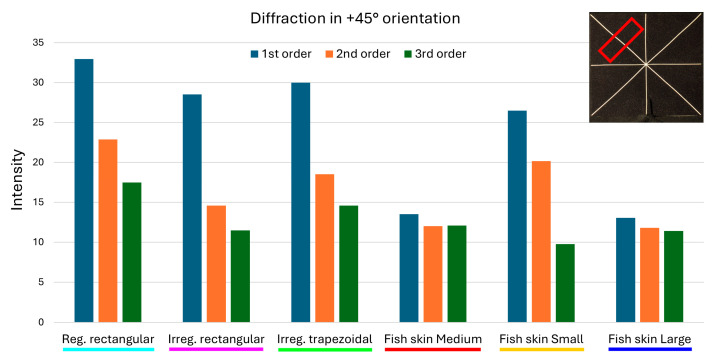
A comparison of the intensities of the first 3 diffraction orders in the +45° direction. The red rectangular shape represents the ROI considered.

The measurements were used to produce clustered column graphs for an at-a-glance comparison of the diffraction intensities produced by the different geometries (Figure 22, Figure 23 and Figure 24).

With regard to diffraction in the 0° direction, the periodic rectangular geometry exhibits an intensity-averaged peak of the first order of 26.64, the non-periodic rectangular of 15.70, and the non-periodic trapezoidal of 21.41, while the *fish skin* Medium geometry exhibits an intensity-averaged peak of 11.33. This means a reduction in the first-order diffraction intensity in the *fish skin* Medium between 57.47% and 27.83% compared to the other arrays similar in aperture size and duty cycle. The *fish skin* Small geometry shows the highest intensity-averaged peak of 29.37, as their aperture dimensions are closer in magnitude to the wavelengths of visible light in contrast to the *fish skin* Large (5.28), which has the largest apertures of the studied geometries. The intensity of second and third orders of diffraction of the *fish skin* Medium (9.92 and 8.99 respectively) are comparable to that of the first order (11.33), and are reduced by 44.86% and 17.82% compared to the equal orders of the periodic rectangular array. The variable aperture spacings and widths of the non-periodic rectangular geometry allow lower second- and third-order diffraction intensities than *fish skin* Medium, resulting in a reduction of 29.94% and 33.93%, respectively.

The analysis of the intensities for diffraction in the −45° and +45° directions confirms the strong reduction in the first-order diffraction produced by the *fish skin* Medium array: 54.33% and 58.62% respectively, compared to the periodic rectangular geometry; 33.74% and 52.59%, respectively, against non-periodic rectangular; and 48.08% and 54.93%, respectively, compared to non-periodic trapezoidal. Second- and third-order diffraction investigation reaffirms the effectiveness of the *fish skin* Medium geometry over the others and yields an additional interesting finding. For diffraction in −45° and +45°, the values of intensities associated with *fish skin* Medium are very close if not better than non-periodic rectangular and *fish skin* Large arrays, exhibiting a percentage difference between the highest and lowest value of 5.34%. This means that the variable aperture spacings and widths and the larger apertures do not provide the same benefits in the second- and third-order reduction seen for diffraction in the 0° direction.

## 5. Fabrication Technology

The micromirror arrays with *fish skin* geometry for smart window applications were fabricated on 10 cm × 10 cm FTO-coated glass substrates NSG TEC™ made by the Pilkington company in our clean room following well-established processes based on techniques involving optical lithography, e-beam evaporation, magnetron sputtering, lift-off, and drying.

The response of the chemical materials and surface technologies used in each step of fabrication to parameters such as temperature, humidity, exposure time, deposition rate, and development time impose proper tuning and optimization. This ensures that each layer of the smart glass achieves the desired characteristics, the array pattern must be transferred efficiently, and the reproducibility must be consistently maintained.

**Figure 25:**
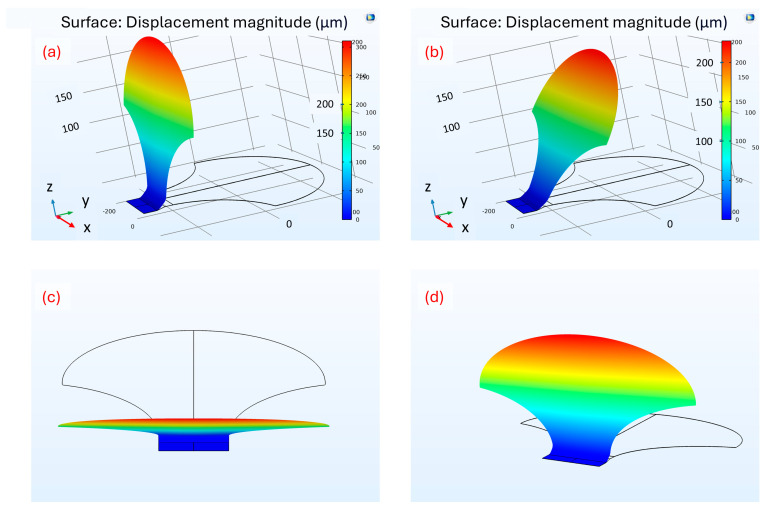
(**a**) Graphic report for micromirror in open position. (**b**) Graphic report for micromirror in intermediate position. (**c**) Image of geometry in open position from top view. (**d**) Image of geometry in intermediate position.

The moving parts of the micromirrors need to exhibit near-perfect planarization to ensure optimal sunlight steering by reflection on them. For this purpose, simulations were carried out with the software COMSOL Multiphysics ver. 5.6 to study the characteristics of planarization along the longitudinal (*L_x_*) and transverse (*L_y_*) length of the micromirror. The physical geometry was imported into the software, and the properties of the materials were assigned to the model. Simulated graphs show that the planarization of the moving part of the *fish skin* micromirror is achievable in all positions, from the initial one with a tilt angle Φ of 90° to its closing, as well as the various positions in between (Figure 25).

**Figure 26:**
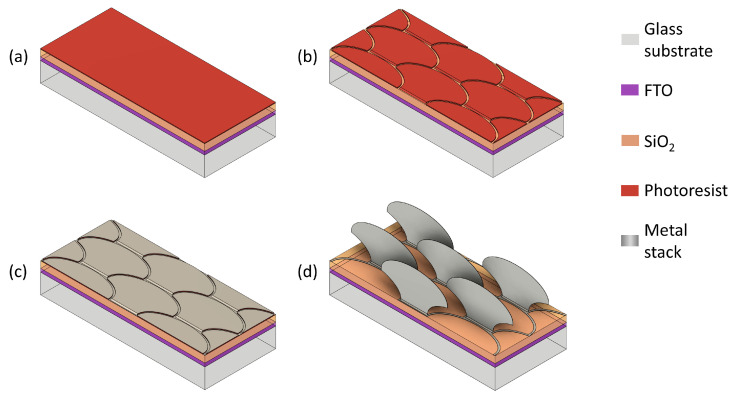
A schematic showing a sample as a result of: (**a**) the deposition of SiO_2_ and photoresist layers on a glass substrate with FTO; (**b**) the *fish skin* pattern after UV exposure and the development of photoresist; (**c**) a metal deposition with stress compensation; (**d**) free-standing planarized *fish skin* micromirrors after lift-off and drying processes.

The glass substrate used has a thickness of 2.3 cm, including a 650 nm thin layer of Fluorine-doped Tin Oxide (FTO) on top serving as a TCO. A SiO_2_ isolation layer of approximately 1000 nm of thickness is then deposited on the FTO glass substrate using the Plasma-Enhanced Chemical Vapor Deposition (PECVD) process [42]. In this fabrication step, a certain area around all the edges of the glass substrate is masked to ensure the contact area of the counter electrode. After cleaning the substrate with chemical and tolerable mechanical treatment, an adhesion promoter is coated by the spin coating method, followed by an activation baking step for 2 min at 120 °C. This improves the adhesion of the sacrificial layer of negative photoresist deposited in the next step, also by the spin coating method (Figure 26a).

Subsequently, a UV photolithography process is executed using a bi-layered photomask developed in our institute, INA, to structure the sacrificial layer [43]. The technology, based on the bi-layered photomask, allows in a single UV exposure step for the creation of the desired leading-edge profile on the straight side of the *fish skin* micromirror structure (representing the anchor with glass surface and the hinge with movable part areas) and the necessary undercut profile on the remaining sides for optimum lift-off [44,45]. After exposure, the sample is immersed in a chemical solution for the development of the photoresist layer (Figure 26b).

Following the first photolithography process, a stack of thin metal films consisting of aluminum (Al) and chromium (Cr) is deposited by electron-beam evaporation in high vacuum conditions, with varying thicknesses in the range of 60–100nm for Al and 10–40 nm for Cr (Figure 26c). The thickness of each functional material is chosen to allow the desired balance between electrostatic attraction and elastic restoration, taking advantage of the inherent stress in the metal stack. An additional photolithography step is executed for selective metal deposition only on the movable areas. Without this additional metal deposition, the micromirrors would stay curled in a free-standing position due to the resulting intrinsic stress in thin metal films, and the desired sunlight steering by reflection would not be possible. The planarization of the micromirror movable parts is achieved by locally compensating the induced residual stress by an additional deposition of the Al layer [46,47,48].

Subsequently, the sample is immersed in a solvent for the lift-off chemical process at high temperatures to remove the sacrificial photoresist layer (Figure 26d).

**Figure 27:**
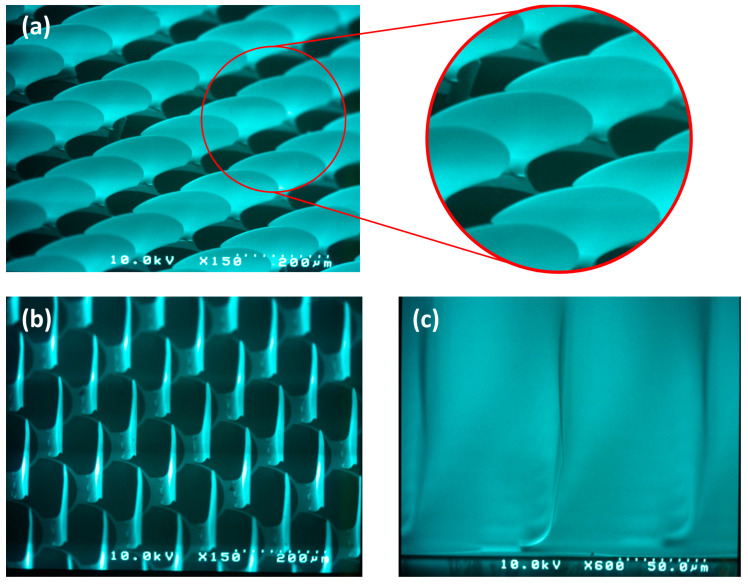
The SEM micrographs of micromirror arrays with *fish skin* geometry: (**a**) good homogeneity over a wide area; (**b**) the planarization of micromirror movable parts; (**c**) a fully open orientation at ~90° to the glass surface.

The released micromirrors are finally dried using a steam-bell technique that exploits a low surface tension. The initial results from the fabrication of 3D arrays with micromirrors inspired by the *fish skin* geometry show promising performance in terms of homogeneity and planarization (Figure 27).

## 6. Conclusions

This study demonstrates the effectiveness of the irregular (non-periodic) rectangular or trapezoidal designs of micromirror arrays in improving the clear view through MEMS smart windows, compared to the conventional regular (periodic) aperture design. The novel *fish skin* geometry is found to be superior compared to all other designs. Fourier transform simulations and optical transmission experiments on different array designs show a significant reduction in non-zero intensity orders of diffraction and a greater homogeneity for the *fish skin* apertures compared to the standard rectangular periodic solution and the corresponding non-periodic configurations. This leads to a substantial improvement of clear view in MEMS smart glasses, opening a promising path in the implementation of light steering systems that operate in transmission through the substrate. Our preliminary work on the fabrication of 3D arrays with micromirrors based on the *fish skin* design shows good results with regard to homogeneity, planarization, and clear view.

## 7. Patent

H. Hillmer: Spiegel-Shutter Array; Patent DE 10 22020 123 024 A1 (2020).

## Data Availability

The original contributions presented in this study are included in the article. Further inquiries can be directed to the corresponding author.
